# The Burden and In-Hospital Mortality of Stroke Admissions at a Tertiary Level Hospital in Namibia: A Retrospective Cohort Study

**DOI:** 10.1155/2023/1978536

**Published:** 2023-02-01

**Authors:** Saara Ndinelago Neshuku, Jessica Kirchner-Frankle, Maria Nangolo, Maria Moses, Chalese Olivia Einbeck, Percy Kumire, Vaja Zatjirua, Justor Banda

**Affiliations:** ^1^Department of Internal Medicine, Division of Neurology, Intermediate Hospital Katutura, Ministry of Health and Social Services, Namibia; ^2^Department of Medical Sciences, School of Medicine, University of Namibia, Namibia; ^3^Department of Internal Medicine, Intermediate Hospital Katutura, Ministry of Health and Social Services, Namibia; ^4^Department of Internal Medicine, Windhoek Central Hospital/Intermediate Hospital Katutura, Ministry of Health and Social Services, Namibia; ^5^Department of Internal Medicine, Division of Neurology, Windhoek Central Hospital/Intermediate Hospital Katutura, Ministry of Health and Social Services, Namibia

## Abstract

**Background:**

Despite stroke being a leading cause of morbidity and mortality globally, there is a dearth of information on the burden and outcomes of stroke in sub-Saharan Africa and Namibia in particular.

**Methods:**

A hospital-based, retrospective cohort study was conducted to analyse non-electronic medical records of all consecutive stroke patients who were admitted to one of the highest tertiary-level hospitals in Namibia for 12 months (2019-2020). The primary outcome of the study was to establish the in-hospital mortality, stroke subtypes, and associated complications.

**Results:**

In total, 220 patients were included in the study, their mean age was 53 (SD13.8) years, and 55.5% were males. 61.0% had an ischaemic stroke (IS), and 39.0% had a haemorrhagic stroke (HS). The mean age was significantly lower in patients with HS vs. IS (48.2 ± 12.2 vs. 56.1 ± 13.3, *p* < 0.001). Of the IS patients, the majority (29.0%) had total anterior circulation infarct (TACI), while in the HS group, 34.0% had basal ganglia haemorrhage with or without intraventricular extension. Hypertension (*p* = 0.015), dyslipidaemia (*p* = 0.001), alcohol consumption (*p* = 0.022), and other cardiovascular diseases (*p* = 0.007) were more prevalent in patients with IS compared to those with HS. The prevalence rate of intravenous thrombolysis was 2.2% in IS and use of intravenous antihypertensives in 25.9% of patients with HS than IS. The in-hospital mortality was 26.4% with complications such as raised ICP, aspiration pneumonia, hydrocephalus, and sepsis significantly high in those that died. Aspiration pneumonia (OR 2.79, 95% CI 1.63-4.76, *p* < 0.001) and increased ICP (OR 0.30, 95% CI 0.16-057, *p* < 0.001) were independent predictors of in-hospital mortality on the multivariate analysis.

**Conclusion:**

Our findings showed a younger mean age for stroke and mortality rate comparable to other low- to middle-income countries (LMICs). Hypertension and alcohol consumption were the main risk factors for both stroke subtypes, while aspiration pneumonia and raised intracranial pressure predicted in-hospital mortality.

## 1. Introduction

Stroke is a leading cause of death and disability worldwide, with an incidence of 11.9 million, a prevalence of 104.2 million and 6.2 million deaths in 2017 [1]. According to the Global Burden of Disease, Injuries, and Risk Factors Study analysis, the overall incidence, mortality, and disability-adjusted life years have declined between 1990 and 2017 [1]. However, this positive trend was more towards high-income countries (HIC) than low- to middle-income countries (LMIC) [2]. Despite a decline in overall stroke incidence, the global burden of stroke remains high, with most cases from LMIC [1]. Despite the poor outcomes associated with stroke, there is a paucity of information in Namibia on prevalence, stroke subtypes, associated complications, and in-hospital mortality.

Previous studies have demonstrated that 80.0% of all new strokes and 87.0% of all stroke-related deaths were in LMIC [1–3]. The absolute incidence and death rates are projected to increase [1], indicating the need to establish stroke prevalence and associated outcomes. Accurate incidence and/or prevalence cannot be determined in the African context because most stroke studies are hospital-based case series [4]. Regardless, these case series still provide valuable information regarding stroke frequency compared to other etiologies of hospital admissions [4].

Undetected and undertreated hypertension remains the leading risk factor for stroke in many developing countries [5]. In a recent study by Nutakki et al., hypertension (80%), heart diseases (34%), and previous stroke (22%) were the most common risk factors identified in Zambia [6]. In another study by Kefale et al., hypertension (64.9%), atrial fibrillation (34.2%), and diabetes mellitus (24.9%) were the common risk factors in northwest Ethiopia [7]. Infectious diseases such as HIV/AIDS burden have also been shown to contribute to the development of stroke in previously published studies [8].

The prevalence of stroke admission was 9.8% in Sierra Leone, with in-hospital mortality of 34.8% [9]. While in Zambia, the in-hospital mortality was 24.0% and 17.0% in northwest Ethiopia, respectively [6, 7].

Namibia is a middle-income country in Southern Africa with a population of more than 2.5 million [10]. In 2013, the prevalence of hypertension, a significant risk factor for stroke, was reported to be nearly 50.0% in Namibia in patients aged between 35 and 64 years [11]. This is expected to increase, especially in urban areas [11].

Similarly, the prevalence of DM was 5.6% in women and 6.7% in men, with a tendency toward the elderly and those residing in urban areas [11].

According to WHO data published in 2018, stroke was estimated to be the third leading cause of mortality in Namibia after HIV/AIDs and cardiac diseases [12]. The prevalence of HIV in Namibia is 8.2%, according to the 2021 spectrum model [13]. Stroke has not been described in Namibia; therefore, we assessed the proportion of stroke admissions, stroke subtypes, risk factors, stroke treatment, and in-hospital outcomes of stroke patients at Intermediate Hospital Katutura, the highest tertiary level hospital in the country.

## 2. Methodology

### 2.1. Study Design, Setting, and Population

**Figure 1:**
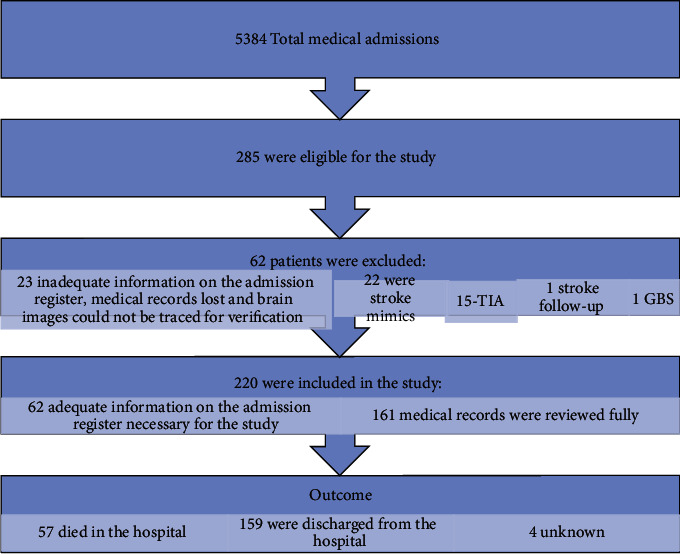
Study flowchart. Abbreviations: TIA: transient ischaemic attack; GBS: Guillain-Barré syndrome.

This is a hospital-based, retrospective cohort review of consecutive nonelectronic medical records of all stroke patients who were admitted to Intermediate Hospital Katutura (IHK) from December 01, 2019, to November 30, 2020. Included in the study were consecutive patients with a new diagnosis of stroke who were aged ≥ 16 years. Censored were patients below 16 years and stroke mimics, transient ischaemic attacks, stroke follow-up and GBS, and medical files of patients with missing information or inadequate information in the inpatient register. A total of 5384 patients were admitted to the medical wards, including the stroke unit, during the study period, and 285 of those patients were eligible for inclusion in the study (Figure 1). The study was approved by the Ministry of Health and Social Services (MOHSS) local ethical committee (Ref 17/3/3/SNN).

IHK is one of the two public tertiary level hospitals in Windhoek, Khomas region. IHK has an 843-bed capacity and is the only public hospital with a dedicated five-bed-stroke unit in Namibia. IHK receives referrals from district hospitals in Namibia's Northeastern, Eastern, Central-Western, and Southern regions of the country. IHK is also one of the leading training centres for medical students from the University of Namibia and runs an internship training program. Three neurologists run the stroke unit with the help of one to two rotating medical officers on a six-monthly basis and two to three medical interns.

The hospital has one CT scanner, which runs 24 hours. All patients with suspected stroke had a CT scan done.

The intravenous tissue plasminogen activators, tenecteplase and alteplase, were available for acute ischaemic stroke patients that presented within 4.5 hours during the study period. However, there were no facilities and expertise for mechanical thrombectomy. The rehabilitation program was limited due to inadequate staffing.

There was one physiotherapist assisted by physiotherapist students, one occupational therapist assisted by one medical rehabilitation worker, one occupational therapist assistant and students, one dietitian, and one speech therapist serving the two public hospitals in Windhoek.

### 2.2. Study Procedures

The inpatient register for the stroke unit, all medical wards, and the acute care unit were reviewed, and patients' information with a stroke diagnosis was extracted. Data extracted were demographic data such as age, gender, marital status, referral pattern, clinical data on length of hospital stay, stroke subtypes, stroke treatment, and vital signs on admission such as blood pressure, temperature, and random blood glucose as well as outcome (death or discharged). Information on ethnicity; comorbidities such as hypertension, diabetes mellitus, and dyslipidemia; alcohol; smoking; cardiovascular diseases including atrial fibrillation; HIV status; history of previous stroke; mental status; Oxfordshire Community Stroke Project (OCSP) classification; haemorrhage location; complications; and rehabilitation status was obtained from the medical records. Nonelectronic medical records were reviewed when information from the inpatient register was missing or there was doubt about the completeness of the admission registry data. Brain imaging was reviewed when the stroke subtype was not specified in the admission register or the stroke location was not specified in the medical records. Blood results, including lipogram, HbA1C, and HIV, were traced from the laboratory when the information was missing from the medical records. The principal investigator (SNN) collected the data from the admission register, nonelectronic medical records, brain imaging, and laboratory investigations with the assistance of three medical officers (MN, JK-F, and COE) using a data collection tool that was created based on the literature review. The data collecting tool was adjusted according to the available data.

### 2.3. Study Outcomes

The primary outcome of the study was to establish the in-hospital mortality, pathological stroke subtypes, and associated complications. The secondary outcomes of the study were to establish the stroke prevalence and to describe age at stroke onset, referral pattern, risk factors, treatment (medical and rehabilitation), and predictors associated with in-hospital mortality.

### 2.4. Standard and Operational Definitions

Stroke was defined according to the WHO as “rapidly developing clinical signs of focal (or global) disturbance of cerebral function lasting >24 hours or until death, with no apparent cause other than a vascular origin” [2, 14]. Hypertension was defined as the current use of antihypertensive drugs or BP > 140/90 mmHg on two recorded readings [14].

DM was defined as the current use of antidiabetic medication or HbA1c of >6.5% or random plasma glucose > 11.0 mmol/L or fasting plasma glucose > 7.0 mmol/L [14]. Dyslipidaemia was defined as the use of cholesterol-lowering agents or documented total cholesterol > 5.2 mmol/L, LDL > 2.5 mmol/L, HDL < 1.0 mmol/L in men or < 1.3 mmol/L in women, and/or triglycerides > 1.7 mmol/L [14]. The previous stroke was defined as a documented history of stroke supported by residual clinical signs and/or CT findings. Smoking and alcohol reported by patients or relatives were noted in the medical records [1, 14]. The OCSP classification into total anterior circulation infarct (TACI), partial anterior circulation infarct (PACI), lacunar circulation infarct (LACI), and posterior circulation infarct (POCI) was used to describe clinical syndromes in patients with ischaemic stroke [15]. Altered mental status was defined as confusion or disorientation, drowsiness reported by family members, or when the attending medical practitioner examined the patient or as it was noted from the onset of the stroke and recorded in the notes [6] or a reduced level of consciousness requiring intubation on admission.

### 2.5. Complication Definitions

A Gugging Swallowing Screen (GUSS) was used to determine the presence and severity of dysphagia and assess the risk of aspiration pneumonia [16]. Malignant middle cerebral artery (MCA) syndrome was defined as rapid neurological deterioration secondary to cerebral oedema from occlusion of the middle cerebral artery territory resulting in an infarct, supported by CT brain findings of midline shift [17]. A haemorrhagic transformation occurred following an acute ischaemic stroke in patients with and without being treated with intravenous thrombolysis and was detected on CT brain [18]. Hydrocephalus refers to abnormal dilatation of the ventricles with resultant raised intracranial pressure following an acute ischaemic stroke or haemorrhagic stroke (intraventricular, intraventricular extension of an existing intraparenchymal hematoma, or subarachnoid haemorrhage) as evidenced by clinical deterioration and confirmed by the CT brain (either initial or a repeat scan) [19]. Raised intracranial pressure (ICP) was diagnosed based on cerebral oedema seen on brain imaging with no hydrocephalus, clinical deterioration, and mannitol treatment as recorded in the medical notes. Aspiration pneumonia was defined as witnessed event of aspiration, a record of empiric antibiotic use when the chest is wet, fever, change in breathing pattern, and/or abnormal chest X-ray [6]. Myocardial infarction was defined by evidence of ischaemic changes on electrocardiogram and positive cardiac enzymes. Pulmonary embolism or deep vein thrombosis was diagnosed by positive findings on CT pulmonary angiogram or venous Doppler flow study.

### 2.6. Data Analysis

Data was transferred from the hard copy to Microsoft excel, coded, and cleaned up. Afterwards, data was imported into the Stata/BE 17.0 program for statistical analysis. Descriptive and inferential statistical analyses were used. Categorical variables were expressed in total numbers, and proportions and associations were compared using Pearson's chi-square test or Fisher's exact test when appropriate. Continuous data were expressed as means ± standard deviation if normally distributed and associations compared with the Student *t*-test.

Skewed data were reported as medians with interquartile range, and comparisons were established with the Wilcoxon rank-sum (Mann–Whitney *U*) test. We employed multivariate logistic analysis using deviations as contrast, backward: LR as a method with probability for stepwise: entry (0.05) with removal (0.051) to determine independent mortality predictors. The level of statistical significance was set at 5%.

## 3. Results

In total, they were 5384 medical admissions during the study period, among which 220 (4.0% prevalence) were stroke admissions. The mean age of stroke patients was 53 (SD ± 13.8) years, and 55.5% were males. Most patients were of African ancestry, accounting for 92.1%. The most frequent underlying comorbidities and risk factors were hypertension, alcohol consumption, dyslipidemia, smoking, and HIV in 86.5%, 58.5%, 51.2%, 39.5%, and 35.0%, respectively. Data for marital status was available in 57.2%, and of these, 53.9% were single, and 40.5% were married. The patients from local urban (Khomas, Windhoek) accounted for about 52.1%, and the rest were referred from district hospitals (Tables 1(a) and 1(b)).

### 3.1. Stroke Subtypes

**Figure 2:**
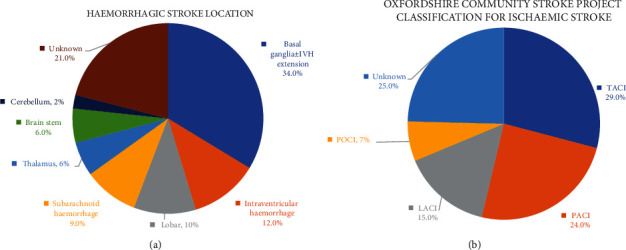
(a) Abbreviations: IVH: intraventricular haemorrhage. (b) Abbreviations: LACI: lacunar infarct; PACI: partial anterior circulation infarct; POCI: posterior circulation infarct; TACI: total anterior circulation infarct.

Of the total 220 stroke admissions, 60.9% (134) had IS, while 39.1% (86) had HS. The mean age in the IS group was significantly older than in HS (56.10 ± 13.9 years vs. 48.24 ± 12.2 years, *p* < 0.001). The patients with HS were more likely to have elevated blood pressure on admission and likely to be intubated at admission (Table 1(a)). In the HS subgroup (Figure 2(a)), 33.7% had basal ganglia bleeding with and without intraventricular extension, 11.6% had an intraventricular haemorrhage, 10.5% had a lobar bleed, 9.3% had a subarachnoid haemorrhage, 5.8% had thalamus bleeding, 5.8% had brainstem haemorrhage, and 2.3% had cerebellum haemorrhage. In the IS subgroup (Figure 2(b)), 29.1% had a TACI, 24.6% had a PACI, 15.0% had a LACI, and POCI was seen in 6.7% of the subgroup.

### 3.2. In-Hospital Stroke Treatment

Only 2.2% (3) of patients with IS received intravenous thrombolysis. During hospitalisation, in the group of stroke patients with hypertension, 43% (77) received three or more oral antihypertensive agents with a trend towards HS than IS (Table 1(b)). In addition, 25.9% (47) of patients required intravenous labetalol or hydralazine to reduce the blood pressure in the acute phase in HS than ischaemic stroke (85.1% vs. 14.9%, *p* < 0.001). In the IS group, 66.1% (82) received aspirin or clopidogrel with a statin, 23.4% (29) received dual antiplatelet therapy and statin, 3.2% (4) received aspirin or clopidogrel, and anticoagulation in 7.3% (9) as secondary stroke prevention (Table 1(b)).

### 3.3. In-Hospital Outcomes and Characteristics of Stroke Survivors Compared to Those That Died

The in-hospital mortality rate was 26.4% (Table 1(b)), and the mortality was significantly higher in the group with haemorrhagic stroke compared to the ischaemic stroke group (52.6% vs. 47.3%, *p* = 0.013). The median time to death was shorter compared to the time to discharge (4.5 days, IQR 1-9 vs. 12.5 days, IQR 6.5-23, *p* < 0.001) (Table 2(b)). On admission (Table 2(a)), the median random blood glucose was elevated in the deceased compared to survivors (7.2 mmol, IQR 5.8-10.8 vs. 6.4 mmol, IQR 5.7-7.9, *p* = 0.048). A large proportion of patients intubated on admission died (63.6% vs. 36.4%, *p* = 0.003). Complications prevalent in the deceased group compared to survivors were (Table 2(b)) raised intracranial pressure (75.0% vs. 25%, *p* < 0.001), malignant middle cerebral artery (MCA) syndrome in ischaemic stroke (90.0% vs. 10.0%, *p* < 0.001), haemorrhagic transformation of ischaemic stroke (66.7% vs. 33.3%, *p* = 0.023), aspiration pneumonia (59.2% vs. 40.8%, *p* < 0.001), hydrocephalus (55.6% vs. 44.4%, *p* = 0.023), and sepsis (52.6% vs. 47.4%, *p* = 0.004). Most patients that received intravenous labetalol or hydralazine and in-hospital physiotherapy survived compared to those that died (62.22% vs. 37.78%, *p* = 0.032 and 94.12% vs. 5.88%, *p* < 0.001, respectively). After adjusting for confounders (Table 3), aspiration pneumonia (OR 2.79, 95% CI 1.63-4.76, *p* < 0.001) and increased ICP (OR 0.30, 95% CI 0.16-0.57, *p* < 0.001) were independent predictors of in-hospital mortality on the multivariate analysis (Table 3).

## 4. Discussion

This study revealed that stroke accounted for 4.0% of all medical admissions and 61.0% had IS, and 39.0% had HS with a younger mean age for stroke onset. Our study also demonstrated that hypertension, alcohol consumption, dyslipidaemia, smoking, and HIV infection were prevalent stroke risk factors, with an in-hospital mortality rate of 26.4%. Aspiration pneumonia and raised intracranial pressure were independent predictors of in-hospital mortality.

The 4.0% prevalence of stroke admission in our study is lower than reported in published studies from rural Kenya (7.09%), Sierra Leone (9.8%), and southwest Ethiopia (16.5%) [9, 20, 21]; however, our findings are consistent with the reports from Gambia (5%) and rural southwestern Nigeria (4.5%) [22, 23]. The lower number of stroke admissions in our cohort might be due to a lack of stroke awareness, poor health-seeking behaviour, and poor referral patterns from district hospitals. Although IHK is the only other public hospital in the country with neurology service and CT scan and the only public hospital with a stroke unit that serves most patients in the country, only 47% of the cohort were referred from other hospitals. At least half of the patients were local referrals.

The mean age in our cohort was 53 years (SD ± 13.8), younger compared to most studies from both developed countries and other sub-Saharan countries (SSA) [6, 9, 14, 20, 24–31]. However, our findings were comparable to other cohort studies from South Africa (53.2 ± 11.4 years), Malawi (54.2 ± 16.9 years), Nigeria (55 ± 15.2 years), and South Ethiopia (53.1 ± 16.9 years) [32–35]. The mean age for stroke patients in the study from Denmark was higher, above 72 years [36]. Therefore, our findings are in agreement with other studies that stroke occurs in relatively younger patients in LMIC than in HIC [9, 24, 25, 27, 32]. The age discrepancies may be attributed to hospital-based studies and age-biased selection in SSA/LMIC; hence, community-based studies are suggested to better describe the exact mean age [9]. In our setting, there is an age-biased referral pattern; younger patients tend to be referred more often than older patients from district hospitals.

IS accounted for 60.9% of the cohort, while HS was 39.1%. Our findings are similar to other hospital-based studies from SSA countries: Rwanda (63.5% IS vs. 36.5% HS), Congo (65.7% IS vs. 34.3% HS), Uganda (64% IS vs. 36% HS), and rural Kenya (67.4% IS vs. 32.6% HS) [20, 24, 31, 37]. A 9-year population-based cohort study from China found that 80% of their patients had IS and 18% had HS (16% ICH and 2% SAH) [38]. A study from Denmark found that 89.9% of patients had IS and 10.1% had HS [36].

The high burden of HS relative to IS in LMIC compared to HIC may be attributed to poor medical care access, poor hypertension control, excessive alcohol use, and smoking [39]. Contributing factors towards HS in our cohort were likely lack of stroke awareness, undertreated hypertension, unavailability of antihypertensive occasionally, and poor compliance to the treatment.

Patients with HS were younger by 8.1 years than IS, consistent with other studies [6, 14, 24, 26, 40]. The differences might reflect different etiologies of each stroke subtype. Contributing to the lower mean age at stroke onset were severe hypertension, hypertensive end-organ damage, alcohol consumption, and HIV-related vascular inflammation [26]. HS is strongly associated with hypertension, the most prevalent stroke risk factor in our cohort. Furthermore, the prevalence of hypertension is also close to 50% in the 35-64 age group in Namibia [11].

Hypertension, alcohol use history, dyslipidaemia, smoking, and HIV were the main risk factors identified in our cohort. This is in agreement with studies performed in South Africa, Zambia, Ethiopia, and Zimbabwe that identified the listed risk factors for stroke despite variations in the proportions of risk factors [6, 41, 42]. The disparity in the risk factor differences could be attributed to different genetic makeup, diet, and lifestyle similarity of each country to HIC [43, 44]. The prevalence of hypertension in our cohort was high, similar to findings from Zambia, Tanzania, Mozambique, Sierra Leone, Ghana, and Nigeria [6, 8, 9, 23, 26, 30, 34].

Alcohol consumption was a significant risk factor for stroke in our study, and this finding is in line with reports from Kenya and Rwanda, in which the rates were 63.0% and 57.1% [20, 37]. This was, however, high compared to most studies in SSA and HIC [44]. A systemic review and meta-analysis by Larsson et al. found that light-moderate alcohol consumption was associated with a low risk of IS, while heavy alcohol consumption was associated with an increased risk of all strokes, but HS more than IS [45]. Alcohol intake is associated with increased high-density lipoprotein cholesterol and fibrinogen levels, explaining the lower risk of IS [45]. However, alcohol intake also negatively impacts blood pressure, increasing the risk of HS. Alcohol is associated with an increased risk of atrial fibrillation, accelerated atherosclerosis, cerebral vasculopathy, and cardioembolism [20].

Dyslipidaemia was the third most common risk factor (51.24%), similar to other SSA studies such as Congo (45.2%), South Africa (47.5%), and Ghana (47%) [24, 30, 41]. Traditionally, Africans have low blood cholesterol compared to Caucasians [24]. The high prevalence of cholesterol, like in other SSA, may reflect changing lifestyles and the adoption of a western diet [24].

Although HIV was the fifth most common risk factor for stroke in our cohort, the prevalence was 35%, higher than in other SSA countries [6, 8, 20, 27, 34, 46]. HIV and stroke can occur coincidentally [47]; however, HIV infection can potentially cause stroke in many possible ways, including indirectly through cardioembolism from HIV-associated cardiac dysfunction, coagulopathy secondary to antiphospholipid syndrome and protein C and S deficiencies, and infective vasculitis from opportunistic infections [47, 48]. The high HIV prevalence in our cohort might reflect HIV infection in the general population. In 2020, Namibia was ranked as the 6th highest rate of HIV in the world [49] and 5th in the SSA between 2003 and 2016 [50].

The risk of stroke in diabetic patients is 2.5 greater than in nondiabetic patients, and early glycemic control is associated with a reduction in strokes [20]. Diabetes mellitus was seen in 20.0% of patients in our study, similar to a report from Sierra Leone [9]. The highest prevalence was recorded in South Africa (86.9%) [41]. A systemic review from Ethiopia reported an 8.0% prevalence of DM [42]. In China, the prevalence of DM was reported to be 6.5% [38].

Only 2.2% of patients with IS received intravenous thrombolysis, which is consistent with the INTERSTROKE study findings [43]. The prevalence of intravenous thrombolysis is low in LMIC compared to HIC, which was 20% [43]. Intravenous thrombolysis is unavailable in most SSA secondary to cost issues [6, 51, 52]. Although acute IS treatment in the form of intravenous thrombolysis is available in our centre, our cohort's low rate of thrombolysis might be explained by the lack of stroke awareness in the community and the failure to recognise a stroke by health workers and the unavailability of brain imaging in peripheral referral facilities and thus delayed referrals.

Antiplatelet therapy with a statin was used in almost all patients with ischaemic stroke, similar to other studies and accordance with IS treatment guidelines [53, 54]. Hypertension was the most common risk factor for both IS and HS; hence, oral antihypertensives were used in most patients, with 43% requiring three or more oral agents. Interestingly, 25.9% of patients were treated with intravenous antihypertensive in the acute phase, and 85.1% had HS. The American Heart Association/American Stroke Association guidelines recommend aggressive lowering of blood pressure in patients with HS when the systolic blood pressure is between 150 and 220 mmHg to prevent hematoma expansion and neurological deterioration [55–57]. According to INTERACT studies 1 and 2, rapid blood pressure reduction to 140 mmHg or less, preferably within six hours, is safe and can improve functional outcomes [56, 57]. The median SBP in our study was 169.5 mmHg, indicating that our treatment with intravenous antihypertensive was in accordance with international guidelines. Unfortunately, the time antihypertensives were given after symptom onset, and the duration of intravenous antihypertensive use was not recorded. And those that required three or more agents to control the blood pressure indicate long-standing hypertension, either undiagnosed, defaulter, or resistant to treatment.

Our study showed that only 52.0% of the patients were referred and seen by the physiotherapist and 94.0% of all patients that received inpatient physiotherapy were discharged home. Studies have shown that a multidisciplinary team consisting of a physiotherapist, occupational therapist, and speech therapist is associated with a better outcome for stroke patients [58, 59]. Early mobilisation in the acute stroke phase and early and intensive physiotherapy reduce morbidity and mortality and improve performing activities of daily living [58, 59]. Only a few patients were seen by the occupational therapist and speech therapist.

The in-hospital mortality was 26.4%, and these findings were comparable with the in-hospital mortality rates from Cameroon, Madagascar, Zimbabwe, Rwanda, and Uganda, which ranged between 23.2% and 30% [14, 27, 31, 37, 60], while the highest in-hospital mortality rate was observed in Gambia, Burkina Faso, Ghana, and Uganda, ranging between 39.1% and 44% [22, 25, 30, 61]. The in-hospital mortality rate was lower in HIC countries such as Germany (4.9%) and Spain (7.13%) [62, 63], and this disparity is due to available resources, stroke units, and advanced stroke care compared to SSA countries where it is estimated that two-thirds of stroke occurs in LMIC [64].

The trend of in-hospital mortality was towards HS at 52.6% (30/57) compared to IS at 47.4% (27/57), similar to Ethiopia, Ghana, Uganda, South Africa, and Denmark [7, 30, 31, 36, 65]. The stroke severity and extent of injury, such as hematoma expansion, oedema, and intraventricular extension, lead to raised intracranial pressure that ultimately contributes to the high in-hospital mortality rate in HS [36, 61]. Although the stroke severity in our cohort was not recorded, most patients requiring intubation also died and thus confirming the theory.

Multivariate analysis showed that aspiration pneumonia and raised ICP were independent predictors of in-hospital mortality in our cohort. In a study from Ghana, 35.0% of deaths were secondary to aspiration pneumonia [30]. In a recent study from Ethiopia, 66.0% of the in-hospital mortality was secondary to aspiration pneumonia, and 16.7% was secondary to raised ICP [51]. Similar findings were observed from the other two centres in Ethiopia [21, 66]. Contributing to the high rate of aspiration pneumonia in our cohort was likely the late presentation to the hospital. The increased median time to hospital presentation after symptom onset, inaccurate assessment of the risk of aspiration by the team, and unavailability of the speech therapist to monitor the swallowing and a physiotherapist for chest rehabilitation are some factors that likely contributed to aspiration pneumonia and poor outcome.

Our study had a significant sample size with several variables that were analysed and revealed an accurate representation of stroke as evidenced by the clinical presentation and supported by CT brain imaging because the cost was not an issue, highlighting the free public health care in Namibia compared to most LMICs; however; it is not without limitations.

Our study had limitations; because it was a hospital-based retrospective study, we cannot rule out bias and account for those who died at home and at the district hospital and patients with minor symptoms who did not present at the hospital. In some patients, alcohol consumption could not be quantified. Recording of information such as the Glasgow Coma Score (GCS), National Institute of Health Stroke Scale (NIHSS), and GUSS was inaccurate and not uniform in all patients, and the modified Rankin score was not recorded; therefore, we could not assess the stroke outcome based on the stroke severity and functional outcome. There was also a significant amount of missing information in the patients' medical records.

## 5. Conclusion

Our findings highlight the stroke burden in Namibia, similar to other LMICs. The mean age was younger, with a trend towards more HS than in HIC as in other LMICs. Hypertension and alcohol were the most risk factors in our cohort. There was a low rate of intravenous thrombolysis and limited rehabilitation. The in-hospital mortality was higher than HIC but comparable to other LMIC. Aspiration pneumonia and raised ICP predicted in-hospital mortality.

Therefore, healthcare workers need to pay particular attention to raising awareness and educating the population on the importance of hypertension treatment, effect of alcohol consumption, and early stroke recognition. Strengthening the importance of early swallowing assessment in every stroke patient is paramount to prevent this complication. Early stroke rehabilitation, including physiotherapy, speech therapy, and occupational therapy, is crucial to improving outcomes.

## Figures and Tables

**(a):** 

	Total (*n* = 220)	Ischaemic stroke (*n* = 134)	Haemorrhagic stroke (*n* = 86)	*p* value
Age (years), mean ± SD	53 ± 13.7	56.1 ± 13.9	48.2 ± 12.2	<0.001
Male (*n*, %)	122 (55.5)	68 (55.74)	54 (44.3)	0.079
Female (*n*, %)	98 (44.56)	66 (67.4)	32 (32.7)	
*Ethnicity*				
African ancestry (*n*, %)	164 (92.1)	95 (57.9)	69 (42.1)	
Mixed ancestry (*n*, %)	8 (4.5)	7 (87.5)	1 (12.5)	
Others (*n*, %)	6 (3.4)	6 (100.0)	0 (0.0)	
*Marital status*				
Single (*n*, %)	68 (53.9)	42 (61.8)	26 (38.2)	
Married (*n*, %)	51 (40.5)	32 (62.8)	19 (37.3)	
Divorced (*n*, %)	1 (0.8)	0 (0.0)	1 (100.0)	
Widower (*n*, %)	6 (4.8)	6 (100.0)	0 (0.0)	
*Referral pattern*				
Khomas (*n*, %)	113 (52.1)	71 (62.8)	42 (37.2)	
Other regions (*n*, %)	104 (47.9)	63 (60.6)	41 (39.4)	
*Vitals on admission*				
(i) Systolic BP (mmHg), median (IQR)	149 (131-177)	143 (128-158)	169.5 (145-196)	<0.001
(ii) Diastolic BP (mmHg), median (IQR)	92 (80-104)	87 (78-98.5)	102 (88-118)	<0.001
(iii) Temperature (°C), median (IQR)	36.5 (36-36.9)	36.5 (36-36.9)	36.5 (35.9-36.9)	0.700
(iv) Random blood sugar (mmol), median (IQR)	6.4 (5.7-8)	6.5 (5.7-8.7)	6.4 (5.7-7.9)	0.806
Altered mental status, yes (*n*, %)	48 (28.57)	25 (52.08)	23 (47.92)	0.078
Intubated on admission, yes (*n*, %)	11 (6.51)	1 (9.09)	10 (90.91)	<0.001
Length of hospital stay (days), median (IQR)	10 (4-18)	11 (6-18)	9 (4-18)	0.209
Duration of Symptoms before admission (days), median (IQR)	1 (0-3)	1 (0-3)	0 (0-3)	0.116

Abbreviations: BP: blood pressure; IQR-interquartile range (Q1-Q3); *n* (%): number (proportion); mean ± SD: mean ± standard deviation.

**(b):** 

	Total	Ischaemic stroke	Haemorrhagic stroke	*p* value
*Stroke risk factors*				
Hypertension, yes (*n*, %)	179 (86.5)	104 (58.1)	75 (41.9)	0.015
Diabetes mellitus, yes (*n*, %)	40 (20.2)	29 (72.50)	11 (27.5)	0.130
Dyslipidaemia, yes (*n*, %)	103 (51.2)	76 (73.79)	27 (26.2)	0.001
Other cardiovascular diseases, yes (*n*, %)	16 (8.3)	14 (87.50)	2 (12.5)	0.007
Smoking history, yes (*n*, %)	51 (39.5)	36 (70.59)	15 (29.4)	0.363
Alcohol history, yes (*n*, %)	79 (58.5)	44 (55.70)	35 (44.3)	0.022
HIV-positive, yes (*n*, %)	28 (35.0)	19 (67.86)	9 (32.1)	0.623
Previous stroke, yes (*n*, %)	14 (8.8)	12 (85.71)	2 (14.3)	0.081
*Medical and rehabilitation treatment*				
Thrombolysis, yes (*n*, %)	3 (2.2)	3 (2.2)	N/A	
Intravenous labetalol/hydralazine, yes (*n*, %)	47 (25.9)	7 (14.9)	40 (85.1)	<0.001
ASA/Clop, yes (*n*, %)	4 (3.2)	4 (3.2)	N/A	
ASA/Clop + statin, yes (*n*, %)	82 (66.1)	82 (66.1)	N/A	
DAPT + statin, yes (*n*, %)	29 (23.4)	29 (23.4)	N/A	
Anticoagulation, yes (*n*, %)	9 (7.3)	9 (7.3)	N/A	
1 antihypertensive agent, yes (*n*, %)	26 (14.5)	16 (61.5)	10 (38.5)	
2 antihypertensive agents, yes (*n*, %)	45 (25.1)	36 (80.0)	9 (20.0)	
3 or more antihypertensive agents, yes (*n*, %)	77 (43.0)	35 (45.5)	42 (54.6)	
Physiotherapy, yes (*n*, %)	85 (51.8)	58 (68.2)	27 (31.8)	0.184
Occupational therapy, yes (*n*, %)	19 (11.6)	11 (57.9)	8 (42.1)	0.595
Speech therapy, yes (*n*, %)	5 (2.6)	3 (60.0)	2 (40.0)	1.000
*Outcome*				
Died, yes (*n*, %)	57 (26.4)	27 (47.4)	30 (52.6)	0.013
Discharged, yes (*n*, %)	159 (73.6)	105 (66.0)	54 (33.9)	0.013

Abbreviations: ASA: aspirin; Clop: clopidogrel; CVS: cardiovascular disease; DAPT: dual antiplatelet therapy; *n* (%): number (proportion); NA: not applicable.

**(a):** 

	Died (*n*, %) 57 (26.4)	Hospital discharge (*n*, %) 159 (73.6)	*p* value
Age (years), mean ± SD	54.91 ± 13.9	52.3 ± 13.6	0.219
Male (*n*, %)	31 (26.3)	87 (73.7)	0.966
Female (*n*, %)	26 (26.5)	72 (73.5)	
Ischaemic stroke (*n*, %)	27 (20.4)	105 (79.6)	0.013
Haemorrhagic stroke (*n*, %)	30 (35.7)	54 (64.3)	0.013
Systolic BP on admission (mmHg), median (IQR)	152 (136-186)	147.5 (131-173)	0.176
Diastolic BP on admission (mmHg), median (IQR)	93 (81-105)	91.5 (80-104)	0.633
Random blood sugar on admission (mmol/L), median (IQR)	7.2 (5.8-10.7)	6.4 (5.7-7.9)	0.048
Altered mental status, yes (*n*, %)	21 (43.8)	27 (56.3)	0.001
Intubated on admission, yes (*n*, %)	7 (63.6)	4 (36.4)	0.003
*Stroke risk factors*			
Hypertension, yes (*n*, %)	49 (28.0)	126 (72.0)	0.052
Diabetes mellitus, yes (*n*, %)	13 (34.2)	25 (65.8)	0.131
Dyslipidaemia, yes (*n*, %)	23 (22.8)	78 (77.3)	0.511
Other cardiovascular diseases, yes (*n*, %)	5 (33.3)	10 (66.7)	0.599
Smoking history, yes (*n*, %)	11 (21.6)	40 (78.43)	0.746
Alcohol history, yes (*n*, %)	16 (20.3)	63 (79.8)	0.680
HIV-positive (*n*, %)	8 (28.6)	20 (71.4)	0.173
Previous stroke, yes (*n*, %)	4 (28.6)	10 (71.4)	0.702
Miscellaneous (*n*, %)	14 (29.2)	34 (70.8)	0.277
Intravenous labetalol/hydralazine, yes (*n*, %)	17 (37.9)	28 (62.2)	0.032
Duration of symptoms before admission (days), median (IQR)	0 (0-2)	1 (0-3)	0.229
Length of hospital stay (days), median (IQR)	4.5 (1-9)	12.5 (6.5-2)	<0.001
Physiotherapy, yes (*n*, %)	5 (5.9)	80 (94.1)	<0.001

Miscellaneous risk factors included current pregnancy, postpartum, increased body mass index, meningitis, tuberculosis, chronic renal failure, COVID-19 infection, trauma to the neck, gout, prostate cancer, herpes ophthalmicus, congenital limb deformity, deaf, epilepsy, asthma, and chronic obstructive pulmonary disease.

**(b):** 

	Died (*n*, %) 57 (26.4)	Hospital discharge (*n*, %) 159 (73.6)	*p* value
*Complications*			
Risk of dysphagia, yes (*n*, %)	26 (38.8)	41 (61.2)	<0.001
Raised ICP, yes (*n*, %)	21 (75.0)	7 (25.0)	<0.001
Malignant MCA, yes (*n*, %)	9 (90.0)	1 (10.0)	<0.001
Hydrocephalus, yes (*n*, %)	10 (55.4)	8 (44.4)	0.023
Depression, yes (*n*, %)	0 (0.0)	7 (100.0)	0.125
Haemorrhagic transformation, yes (*n*, %)	4 (66.7)	2 (33.3)	0.023
Aspiration pneumonia, yes (*n*, %)	29 (59.2)	20 (40.8)	<0.001
Acute kidney injury, yes (*n*, %)	12 (38.7)	19 (61.2)	0.052
Urinary tract infection, yes (*n*, %)	4 (14.3)	24 (85.7)	0.183
PE/DVT^∗^, yes (*n*, %)	1 (50.3)	1 (50.0)	0.443
Myocardial infarction^∗^, yes (*n*, %)	2 (100.0)	0 (0.0)	0.061
Hyponatremia, yes (*n*, %)	5 (23.8)	16 (76.2)	0.984
Sepsis, yes (*n*, %)	10 (52.6)	9 (47.4)	0.004
Miscellaneous, yes (*n*, %)	9 (60.0)	6 (40.0)	0.001

Abbreviations: CVS: cardiovascular disease; ICP: intracranial pressure; MCA: middle cerebral artery; PE/DVT: pulmonary embolism/deep vein thrombosis. Miscellaneous complications included persistent hyperglycemia/hypertension, hyperkalemia/hypokalemia, hypernatremia, anaemia, obstructive sleep apnoea, pulmonary oedema, and HELLP syndrome. ^∗^Fisher exact test.

**Table 3:** Univariate and multivariate analyses of the in-hospital mortality.

Characteristics	Unadjusted odds ratio (95% CI)	*p* value	Adjusted odds ratio (95% CI)	*p* value
SBP	1.01 (1.00, 1.01)	0.079	—	
HGT	1.16 (1.03, 1.31)	0.018	—	
Raised ICP				
Yes	4.18 (2.57, 6.79)	<0.001	0.30 (0.16, 0.57)	<0.001
No	1			
Hydrocephalus				
Yes	2.13 (1.29, 3.51)	0.003	—	
No	1			
Haem transformation				
Yes	2.43 (1.03, 5.77)	0.043	—	
No	1			
Asp pneumonia				
Yes	3.25 (2.19, 4.81)	<0.001	2.79 (1.63, 4.76)	<0.001
No	1			
AKI				
Yes	1.50 (0.99, 2.26)	0.055	—	
No	1			
Sepsis				
Yes	1.96 (1.20, 3.19)	0.007	—	
No	1			

Abbreviations: SAH: subarachnoid haemorrhage; ICP: intracranial pressure; SBP: systolic blood pressure; Haem transformation: haemorrhagic transformation; AKI: acute kidney injury; Asp pneumonia: aspiration pneumonia.

## Data Availability

The data supporting the findings of this study are included within the article.
